# Research on cavitation effect of microtextured array

**DOI:** 10.1038/s41598-022-17258-0

**Published:** 2022-08-04

**Authors:** Yuanyuan Jiang, Zhijun Yan, Shengwei Zhang, Ziyu Shen, Haocheng Sun

**Affiliations:** Marine Engineering College, Dalian Maritime University, Dalian, Liaoning 116026 China

**Keywords:** Engineering, Materials science

## Abstract

In this paper, the surface texture parameters and distribution patterns are studied by numerical simulation and experiment. First, a three-dimensional micro-textured CFD fluid lubrication model with cavitation effect is established, and different texture arrays are designed to study the influence of different distribution modes on bearing capacity, friction coefficient and pressure distribution of the oil film. Then, the simulation results are further analyzed and verified by the visualized plane slider experimental platform, and the formation rules of cavitation bubbles in the micro-textured array, as well as the influences of the surface shape and different distribution modes of the micro-textured array on the cavitation bubbles are discussed. The results show that the existence of cavitation is one of the main reasons for the microtexture to increase the bearing capacity of the oil film, which cannot be ignored in the simulation study. The texture array with single symmetric orientation is the best to improve the oil film bearing capacity, and the bearing performance is the best when the texture inclination angle is 26.6°.The friction coefficient of the asymmetrically oriented textured array is 29.4% lower than that of the non-textured sample.The results in the experiment are consistent with the simulation.

## Introduction

Surface texturing, which generally use a specific processing technology to prepare the microstructure with a certain size, shape and arrangement on the surface of the friction pair, can be applied to improve the hydrodynamic lubrication performance of mechanical components^1^. Surface texture with a reasonable design may offer significant improvement Under fluid lubrication conditions, the cavitation effect^2–4^ induced by texture can significantly increase the bearing capacity^5,6^ of the friction pair. Therefore, the theoretical and experimental research on the texture induced cavitation effect is very important to improve the lubrication^7–9^ of mechanical parts and reduce energy consumption^10^.

The geometric parameters of microtexture are the main factors affecting the hydrodynamic lubrication performance of surface texture. A suitable design of geometric parameters can make the textured surface exhibit the optimal lubrication and friction reduction performance^11,12^. Wang et al.^13^ fabricated circular microtextures on the thrust bearing surface by reactive ion etching, and conducted a series of experimental studies on the load-carrying capacity of micropits with different sizes, depths, and densities under water lubrication conditions. Yu et al.^14^ investigated the effect of different dimple shapes (circular, triangular, and elliptical) and the arrangement direction of textures on the pressure distribution. While Nanbu et al.^15^ focused on the optimization of the bottom surface topography of craters. Wang et al.^16^ designed the surface texture of SiC surface with different distribution modes, and the results show that the mixed texture can improve the load-bearing capacity of SiC ceramics more than the texture of a single size. Shen et al.^17^ proposed a numerical texture optimization method based on the SQP algorithm.

In terms of numerical simulation studies, Siripuram and Stephens^18^ conducted numerical analysis for different shapes of crater and convex body weaves. Caramia et al.^19^ solved the micro-texture surface lubrication performance analysis of the Reynolds equation and the N-S equation for the microloom respectively, and in comparison found that the inertial force has a significant effect on the dynamic pressure effect of the microloom and is an important cause of fluid Liu et al.^20^ established a two-dimensional model of asymmetric microtexture, and studied the influence of the outlet inclination angle of the texture on the fluid pressure distribution, flow field form, upper wall bearing capacity and friction coefficient in the textured area. Jiang et al.^21^ used ANSYS Fluent to establish a numerical model of the three-dimensional flow field of a mechanical seal with an elliptical loom. The difficulty of solving the N-S equation has been greatly reduced due to the current improvement in computer performance and the popularity of commercial CFD codes based on the N-S equation^22^. Therefore, it has become an inevitable trend to use the N-S equation to solve the weaving lubrication model considering the cavitation effect.

For surface weave cavitation experiments, a visualization bench is needed to directly observe the cavitation phenomenon^23,24^ and study the formation mechanism. Qiu et al.^25^ experimentally observed the cavitation phenomenon of surface craters in thrust bearings, studied the effect of velocity on the cavitation effect, and suggested that velocity may affect the cavitation pressure.Reiner Wahl et al.^26^ used pin-disk experiments to observe the mesh groove Weaving surface-induced cavitation phenomenon, found that bubbles existed as small tails at the downstream end of the convex body or around the sides of the convex body, and found that a certain velocity must be reached for cavitation to occur. Bai et al.^27^ investigated the transient growth process of microweaving-induced lubricant-generated cavitation phenomenon on the surface of a thrust bearing and found that the bubbles in the cavitation region increased with the increase of running time and gradually reach a steady state, which implies that a transition period is required to reach equilibrium. It is also concluded that the air precipitated from the lubricant is the main component of the cavitation bubbles and the evaporation of the lubricant has little effect.

In summary, the above literature has less research on the effect of synergy between the weave arrays, as well as few three-dimensional CFD models have been established considering cavitation, and the morphology of the array weave is relatively single. Secondly, in terms of experiments on the induced cavitation of woven arrays, the experiments related to the distribution mode of woven arrays are also less involved in the literature.

The purpose of this study is to explore how the micro-textured array regulates the occurrence of cavitation. “Result” Section describes the establishment, solution and analysis of the micro-textured array model. Considering the cavitation effect, three-dimensional numerical simulation of the surface microtexture is carried out by using ANSYS-Fluent software to study the influence of texture distribution modes on the lubrication performance.In order to verify the rationality of the simulation results, the corresponding experiments are carried out by a visual plane sliding platform to analyze the formation of cavitation bubbles. Experimental details, analysis and results are presented in “Discussion” and “Conclusions” Sections. Finally, the conclusions are provided in the last section.

## Result

### Simulation of micro-textured arrays

#### Geometric model

**Figure 1:**
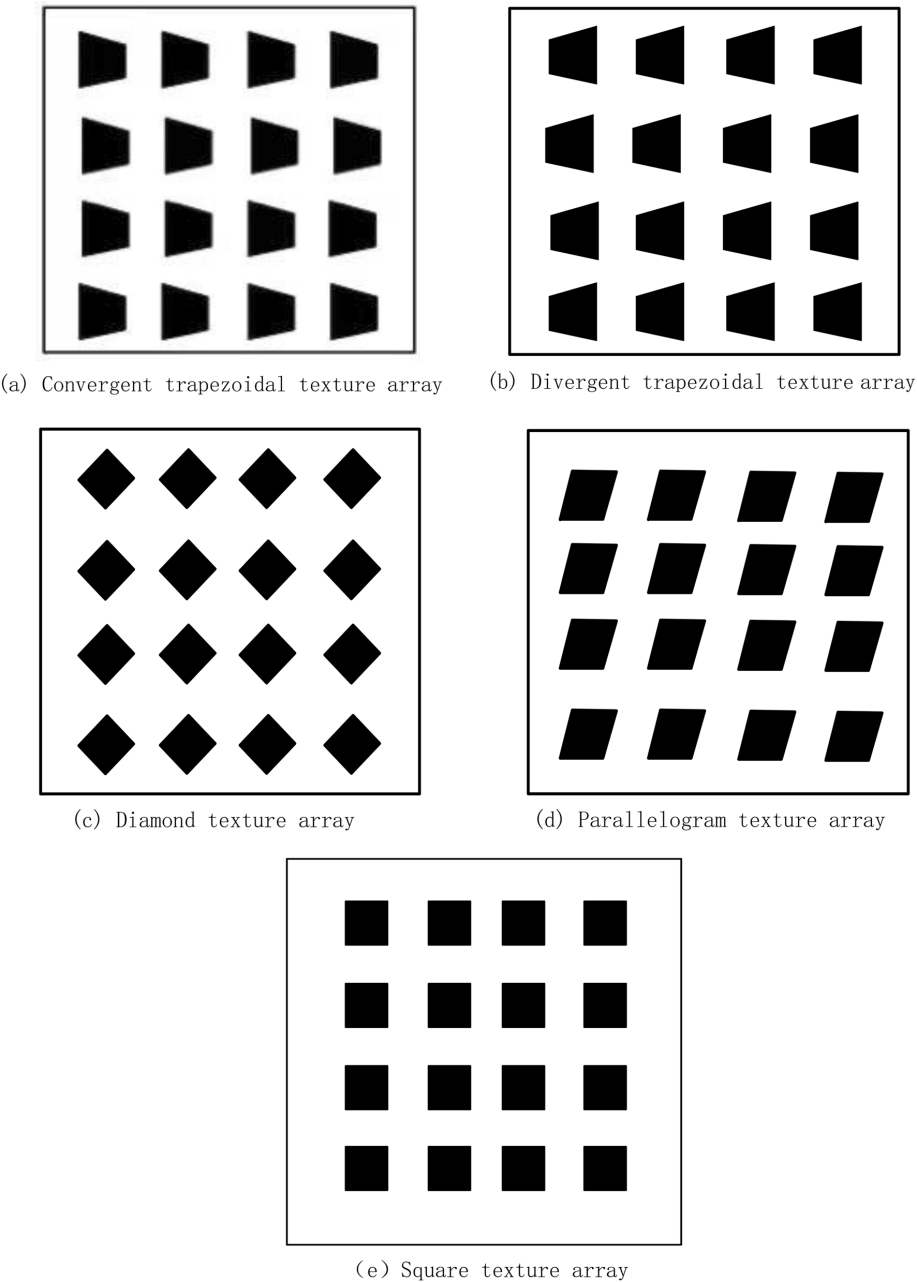
Five types of 4 × 4 microtexture array layout.

When studying the influence of micro-texture on the fluid lubrication effect, most scholars choose to simulate the flow field of a single texture, but ignore the mutual influence between textures. The cooperation relationship between the texture is not clear yet, which is worthy of further study. Therefore, this study establishes a 4 × 4 micro-texture array model as shown in Fig. 1 for simulation calculation analysis. First of all, the 4 × 4 microtexture array model can better analyze the variation law of its pressure, bearing capacity and friction force in the horizontal and vertical directions. Secondly, selecting a 4 × 4 micro-texture array model can better perform simulation calculations. Overly complex structural design will lead to a sharp increase in the amount of calculation, which is not conducive to analyzing data.

#### Numerical validation

In this study, the ANSYS-Fluent three-dimensional double-precision solver is adopted for simulation calculation, and *k-ε* turbulence model. The cavitation simulation uses the Mixture multiphase flow model and the Schnerr-Sauer cavitation model. The pressure-velocity coupling uses the Coupled method, the momentum term and energy term are both selected as Quick, the pressure term is in the *PRESTO!* format, and the convergence factor is 1 × 10^−5^. In order to facilitate the calculation, the remaining conditions are chosen as Default. The solution process mainly includes the establishment of the geometric model and the meshing, the setting of boundary conditions, physical parameters and the solution method, and the post-processing analysis with *Tecplot* software. The physical parameters used in the simulation are shown Table 1.

**Table 1:** Parameters for the numerical experiments.

Calculation parameters	Numerical value
Atmospheric reference pressure	101,325.1 *p*_0_/Pa
Cavitation pressure	30,000 *p*_c_/Pa
Lubricating oil viscosity	0.098*η*/(Pa·s)
Lubricant density	891*ρ*/(kg·m^-3^)

In order to compare the effects of different texture models on the lubrication performance, dimensionless bearing capacity, dimensionless friction and friction coefficient *f* are selected to characterize the lubrication performance of the friction pair:

1 $$F_{y} = \iint {p(x,z)dxdz}\;\;\;\;\overline{{F_{y} }} = \frac{{F_{y} }}{{F_{0} }} = \frac{{F_{y} }}{{p_{0} S_{d} }}$$

2 $$F_{x} = \iint \tau ^{\prime}dxdz\;\;\;\;\overline{{F_{x} }} = \frac{{F_{x} }}{{F_{0} }} = \frac{{F_{x} }}{{p_{0} S_{d} }}$$

3 $$f = \frac{{F_{x} }}{{F_{y} }}$$

In the formula, *F*_*y*_ is the normal bearing capacity, *p(x, z)* is the pressure distribution on the upper wall, *F*_*0*_ is the reference force, *p*_*0*_ is the atmospheric pressure, *F*_*x*_ is the tangential friction force, and *τ′* is the shear force.

### Experiment

In the section , in order to verify the occurrence and behavior of cavitation induced by the micro-textured array, the lubrication test and observation of texture samples were carried out on a self-made visual tribo-tester.

#### Experimental apparatus and specimen preparation

**Figure 2:**
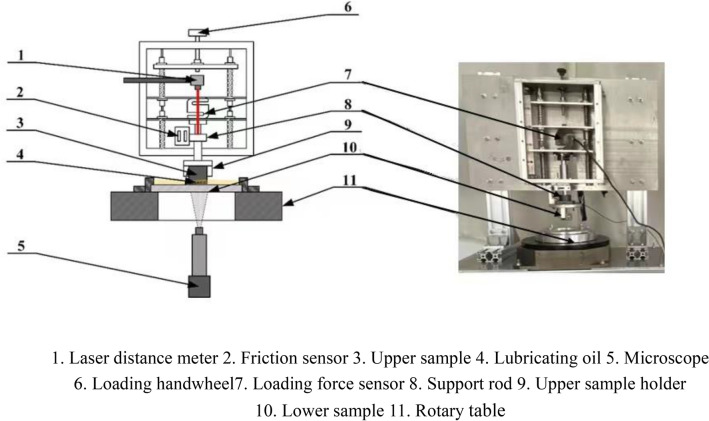
Sample and fixture assembly drawing.

Figure 2 shows a schematic diagram of the visual flat tribo-testers. The upper sample is tightened by the clamp(9), and the lower sample is connected to the rotating unit which is driven by a stepping motor, and lubricating oil is added between the samples. The vertical load is applied to the support rod by the loading handwheel(6). The loading force is measured by the pressure sensor 7, and the friction force is measured by the pressure sensor 2. A microscope is used to observe through the glass sample (10) from below and to take cavitation image.In this experiment, in order to facilitate preparation and observation, the upper sample is made of brass; and it is designed into a cylindrical shape with rectangular section of 1.2 mm × 2.0 mm to facilitate installation and match with the lower sample.

#### Experimental procedure

In this experiment, Castrol 5 W-40 lubricanting oil is used as lubricant. The kinematic viscosity is 86 mm^2^/s (40 ℃) and 13.7 mm^2^/s (100 °C) respectively, and its density at room temperature (25 °C) is 0.856 g/cm^2^. The experiment is carried out at room temperature, the dynamic viscosity of the lubricanting oil at room temperature is 0.0933 Pa·s. Before the experiment, sufficient lubricating oil is added in the oil tank of the rotating disc, and the textured sample is immersed into the lubricating oil. The tribo-tester needs to operate under no-load, low speed and sufficient lubrication for a period of time, so that a small amount of air stored in the texture pit can flow out with the lubricating oil. When there are no more bubbles in the texture, stop the rotating unit, reset the test load, speed and other parametersand , and restart the rotating unit for formal test. The final test results are obtained through measuring frictiona and other related parameters under stable conditions, and monitor and photographing the cavitation images of the friction surface in real time through an image acquisition system.

## Discussion

### Analysis of simulation

#### The effect of texture arrays of different shapes

**Figure 3:**
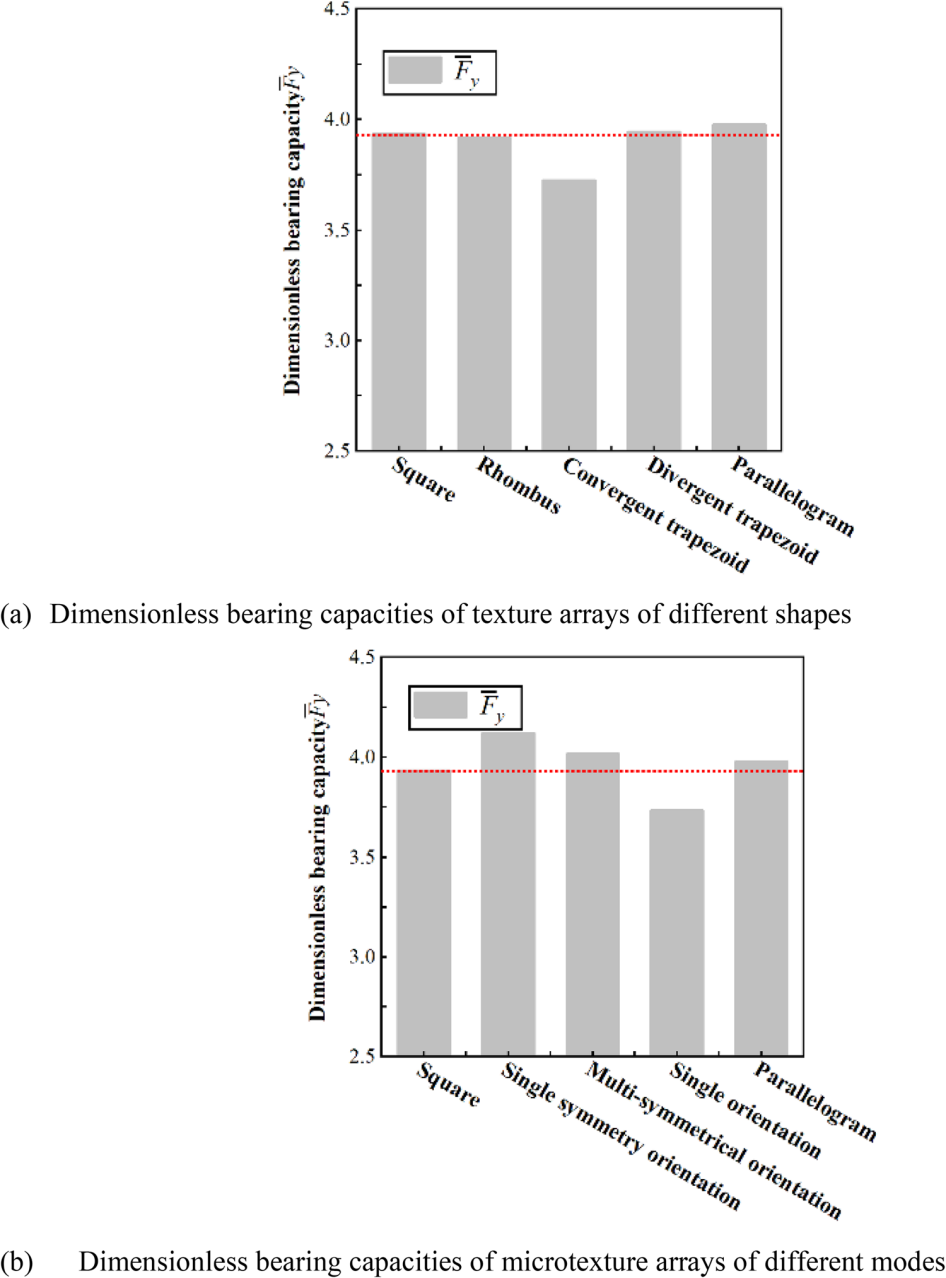
Dimensionless bearing capacities.

**Figure 4:**
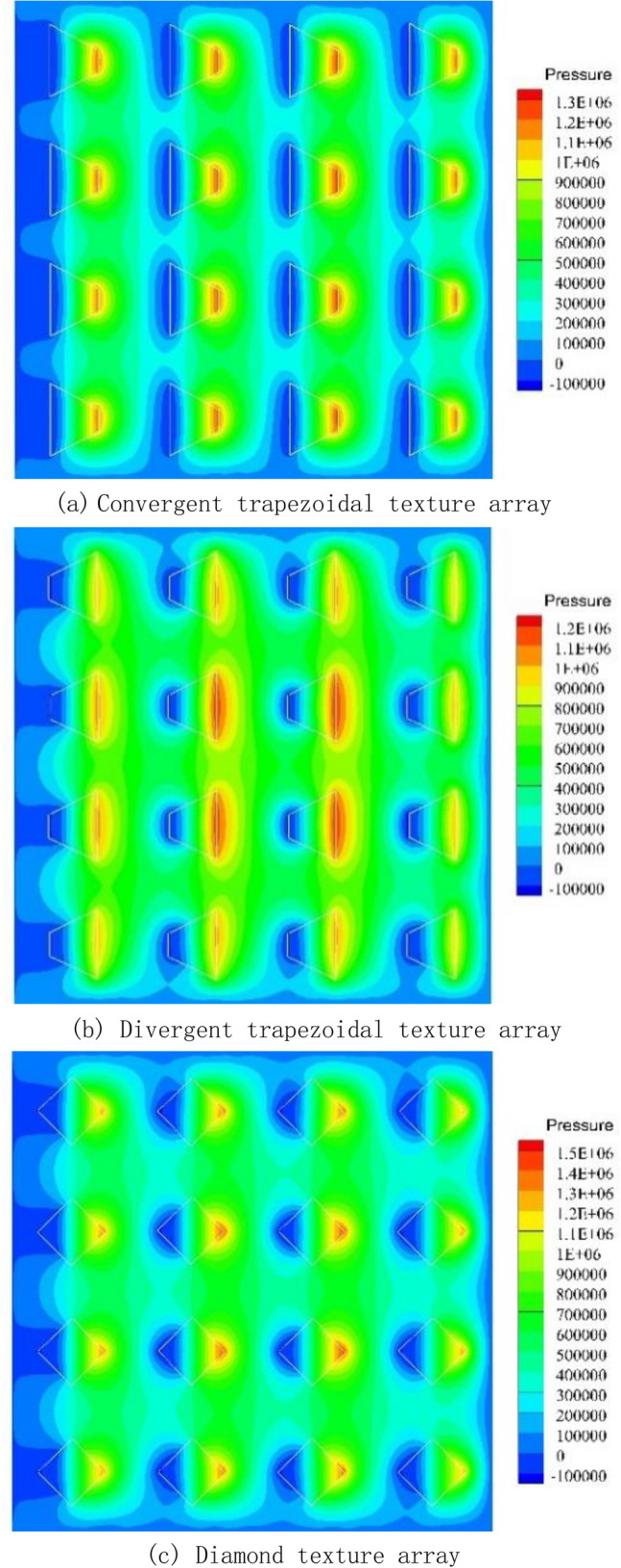

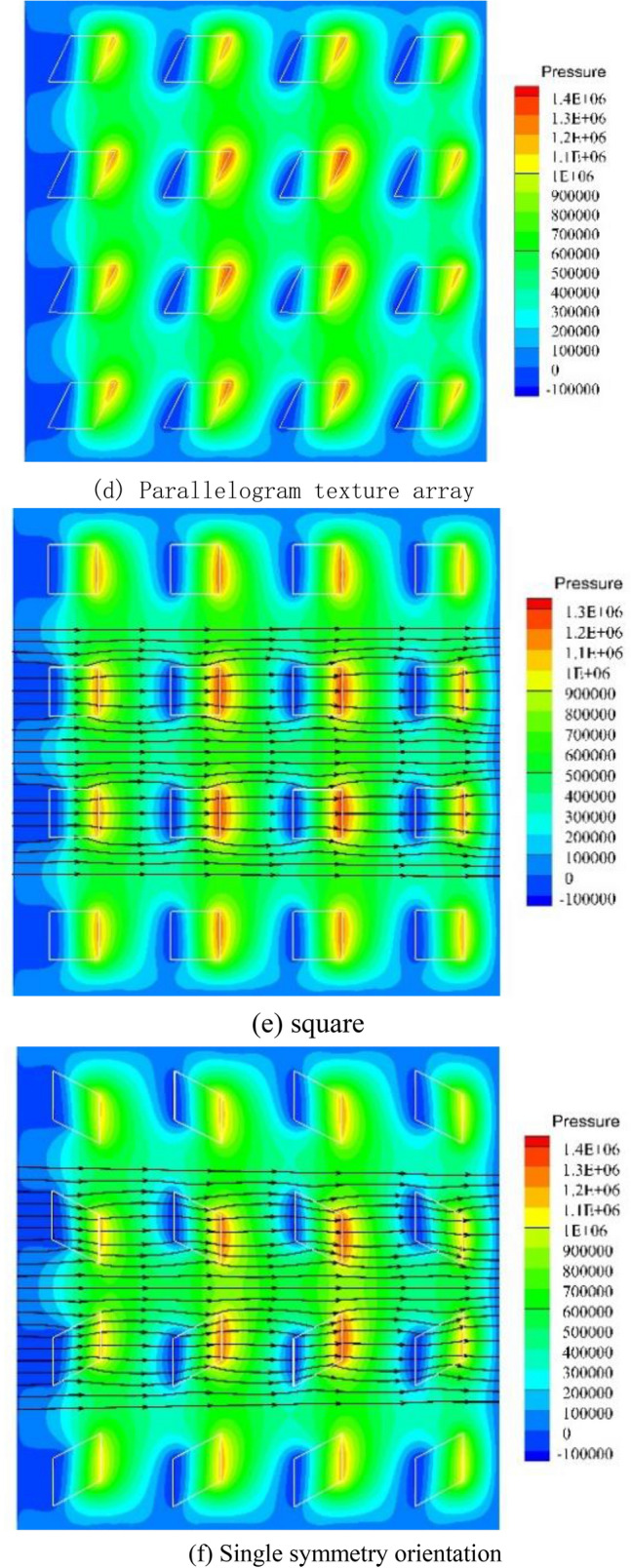

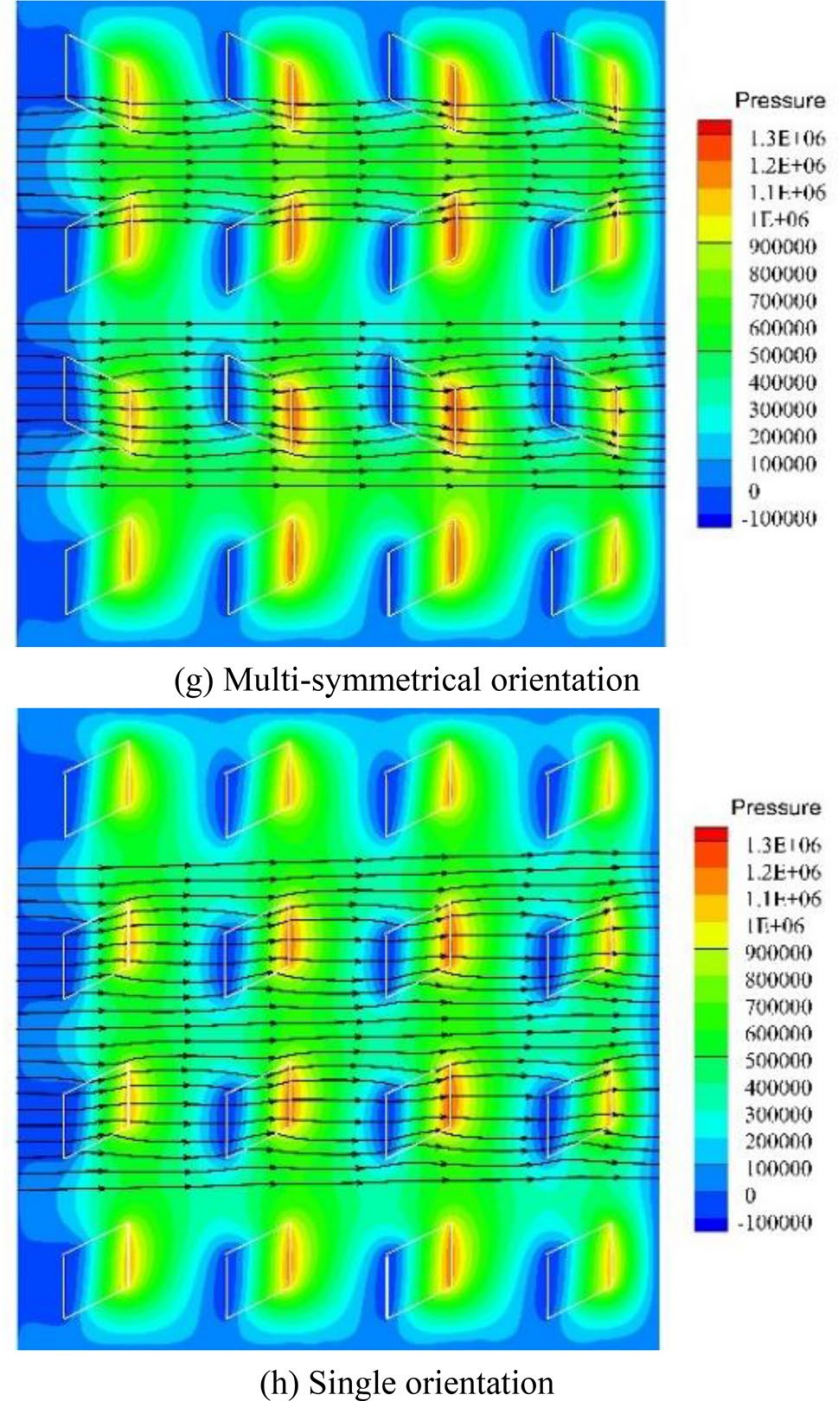
Pressure distribution.

Figure 3a shows the dimensionless bearing capacity of a texture array of different shapes at a speed of 6 m/s, the specimen with a parallelogram texture array has the maximum bearing capacity. Figure 4 shows the pressure distribution on the upper wall of the texture array with different shapes. From Fig. 4a,b, it can be concluded that due to the confluence role of textures, the maximum pressure of the convergent trapezoidal texture array is greater than that of the divergent trapezoidal texture array, but the area of the boost zone is much smaller than that of the divergent trapezoidal texture array, which eventually leads to lower total bearing capacity for the sample with convergent trapezoidal texture array.The reason is that for the texture array the mutual influence of adjacent textures perpendicular to the motion direction needs to be considered. Therefore, it can be concluded from Fig. 4c, d, the highest pressure of the diamond array texture is greater than that of the parallelogram, but the high pressure peak area and low pressure cavitation area of the parallelogram have both shifted. So the inhibition between the front and rear microtextures is reduced, and the overall bearing capacity is enhanced, which is better than that of the rhombic texture array, which is the same as the simulation result of single texture.

#### Influence of parallelogram textured array distribution mode

As shown in Fig. 3b, the dimensionless bearing capacity of five different distribution patterns of micro-textured arrays at speed of 6 m/s is compared.From the figure,it can be seen from the figure that the matching mode between the micro-textures has a stronger influence on the flow field and pressure field in the numerical simulation than simply changing the shape of the textures.

Figure 4e–h show the pressure distribution of the square micro-textured array and the three different pattern arrays. Different patterns of parallelogram texture arrays will not only change the pressure field of the fluid domain but also affect the flow field. From the streamline diagram of the square texture array, it can be seen that the streamlines are only partially bent near the texture entrance and exit, and the adjacent textures will not affect their respective flow fields. In the single-symmetric and pair-symmetric texture arrays, the inclination of the parallelogram texture makes the flow field appear obvious confluence effect, which is conducive to the production of higher oil film pressure. The streamlines in the texture array with single orientation are obviously inclined, but there is no effect of confluence, and the lateral flow of the fluid will cause energy dissipation, weaken the dynamic pressure effect of the texture, and make it the lowest carrying capacity.Similar to the parallelogram texture in the single-texture simulation, the texture arrays of other different distribution patterns also have the effect of reducing the suppression effect between the front and back textures, because of the high-pressure peak area and the low-pressure cavitation area have shifted. The high-pressure area of the outlet can better extend to the direction of fluid flow.

#### Influence of the tilt angle of the parallelogram texture array

**Figure 5:**
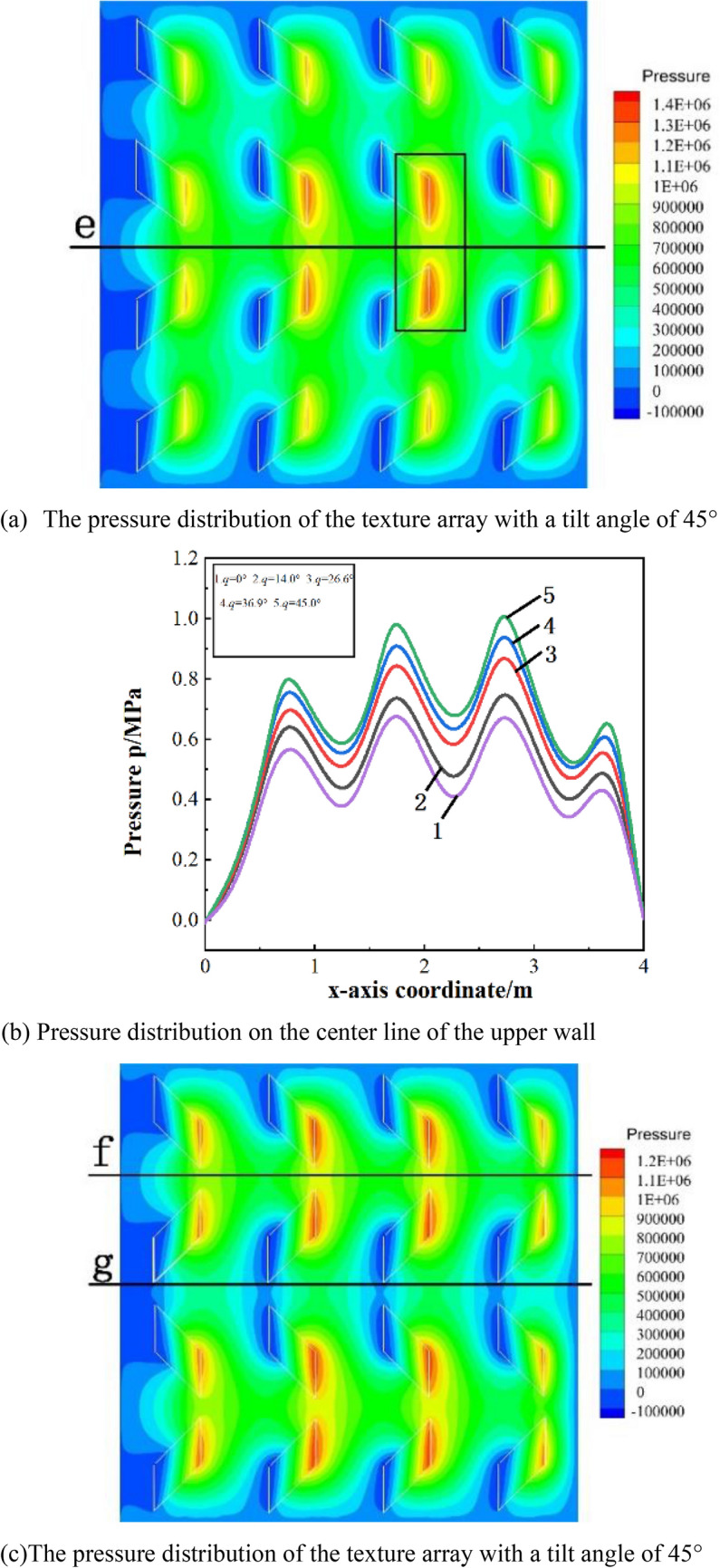

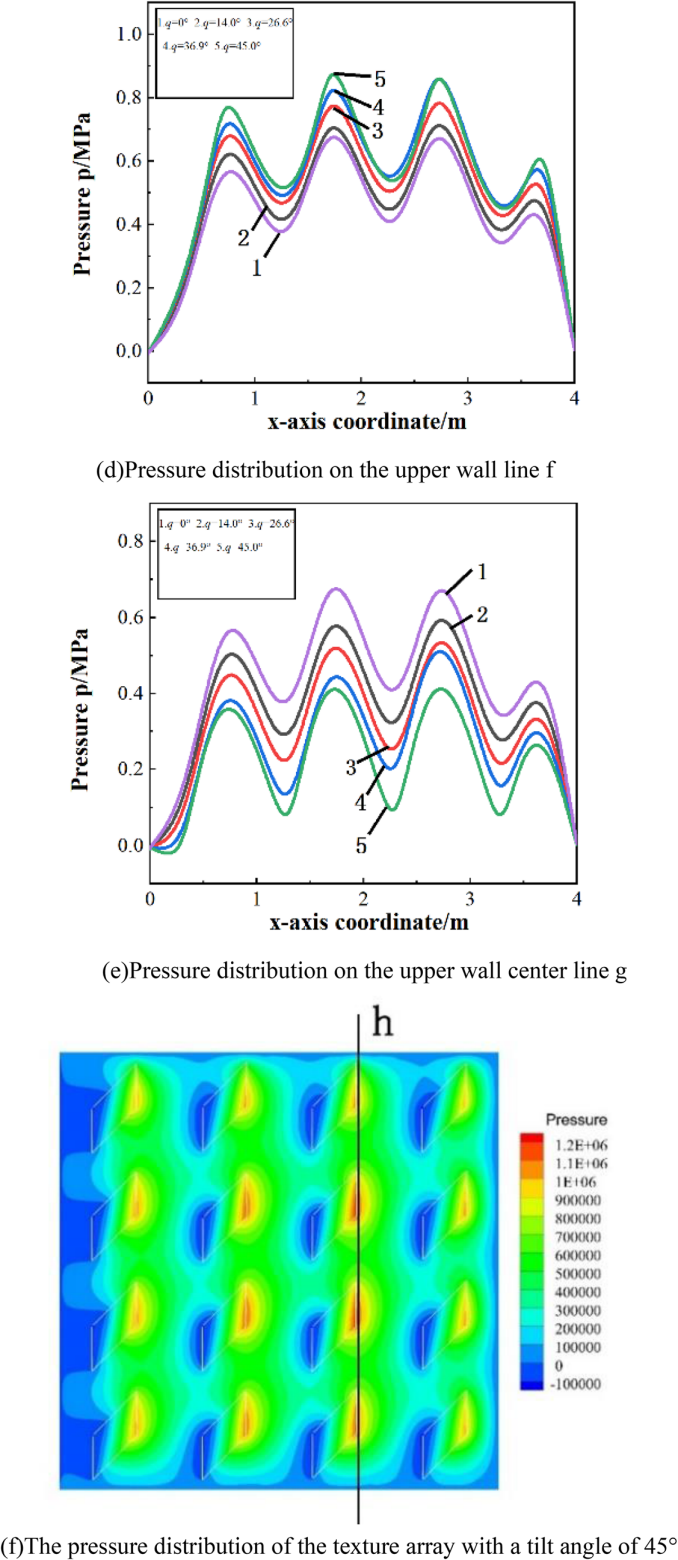

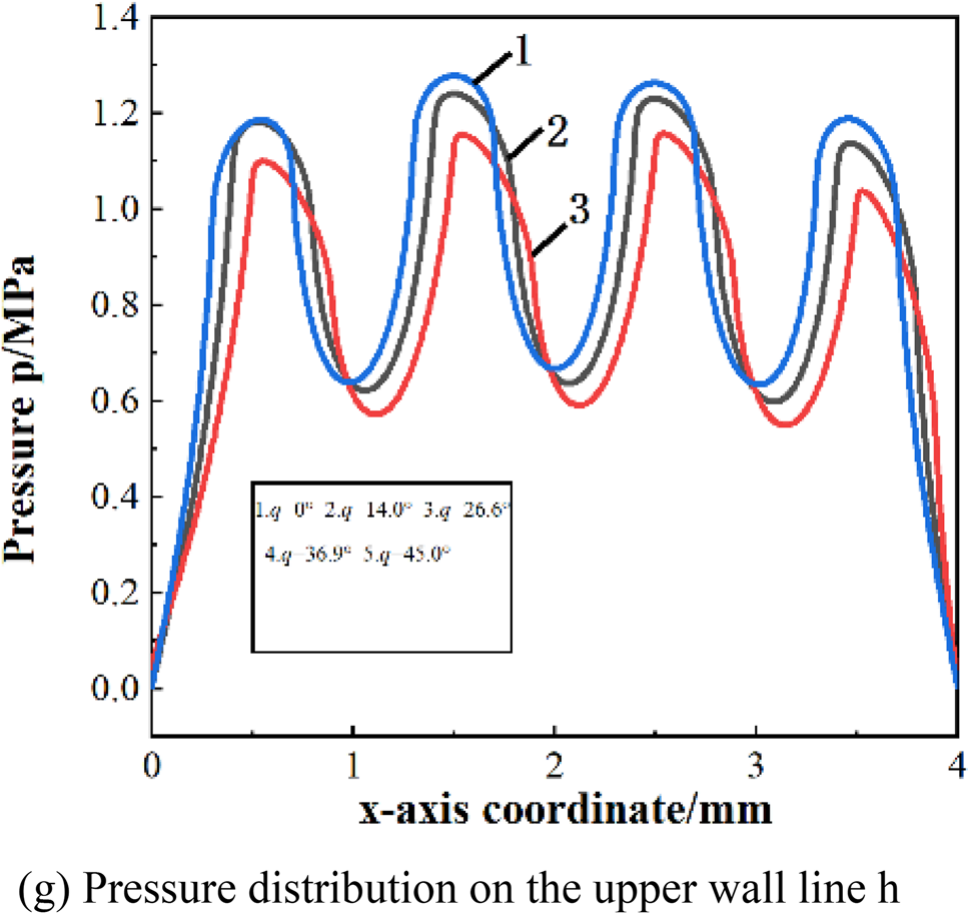
Pressure distribution with different tilt angles.

Figure 5a shows the pressure distribution of the texture array with a single symmetry orientation. As shown in Fig. 5b, in a single symmetry orientation texture array, as the tilt angle increases, the pressure on the center line e of the upper wall increases significantly. With the increase of the inclination angle of the microtexture, the outlet pressurization zone of the second and third rows of microtexture will be closer, which improves the oil film pressure. However, if the inclination angle of the micro-texture is too large, it will reduce the range of the pressurized zone and affect the Oil film pressure.

Figure 5c shows the pressure distribution of a multi-symmetrical orientation texture array. As shown in Fig. 5d, the oil film pressure on the straight line f between the first and second rows of textures will gradually increase as the tilt angle increases. In addition, it can be seen from Fig. 5e that the increase in the inclination angle of the textures will reduce the pressure on the center line g of the upper wall surface.

Figure 5f shows the pressure distribution on the upper wall of the textured array with a single orientation when the tilt angle is 45°. It can be concluded that a texture array with a single orientation cannot converge pressure.Take the straight line h at the exit of the third row of textures to analyze the pressure distribution ,as shown in Fig. 5g. As the tilt angle increases, the pressure peak at the exit of the parallelogram texture shifts to the tilt direction, and the pressure also reduce accordingly at the same time. Therefore, it is believed that parallelogram micro-textured array with single orientation has no effect on improving the bearing capacity compared to a square texture, and the larger the tilt angle, and the increase of the tilt angle will weaken the dynamic pressure effect of the texture.

#### Influence of the speed of the upper wall

In^28^, the existence of eddy current was found by analyzing the velocity field, and the following explanation was given: the increase of the depth of the micro-dimples will enhance the wedge effect and improve the hydrodynamic pressure performance, but on the other hand, the eddy current phenomenon will lead to energy dissipated, impairing the hydrodynamic bearing capacity. Therefore, there will be an optimal value for the texture depth, so that the dynamic pressure bearing performance of the micro-pit unit is the strongest.This indicates that the weakening of the hydrodynamic performance with the increase of texture depth is due to the combined effect of the reduction of the cavitation zone and the increase of the vortex zone.

**Figure 6:**
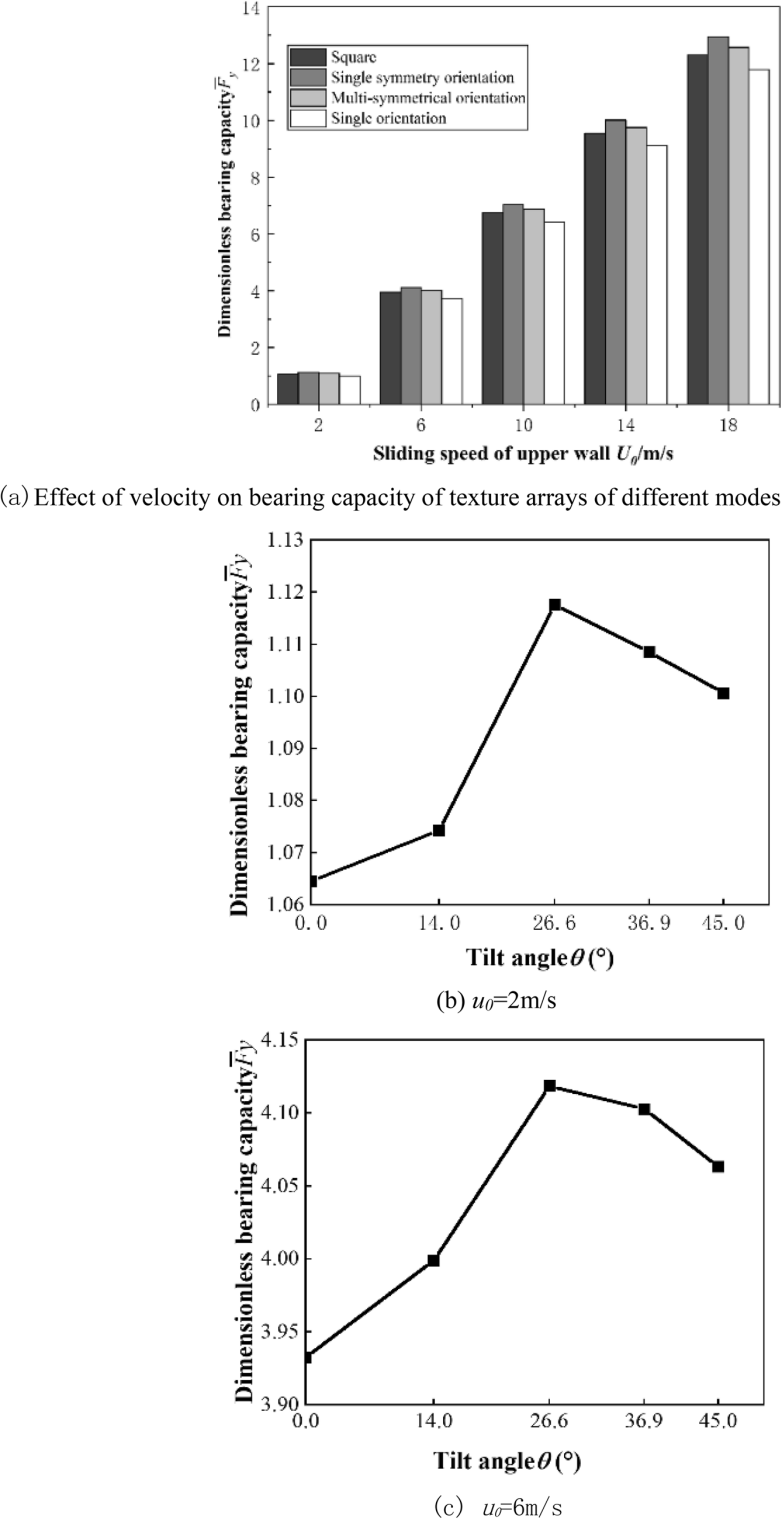

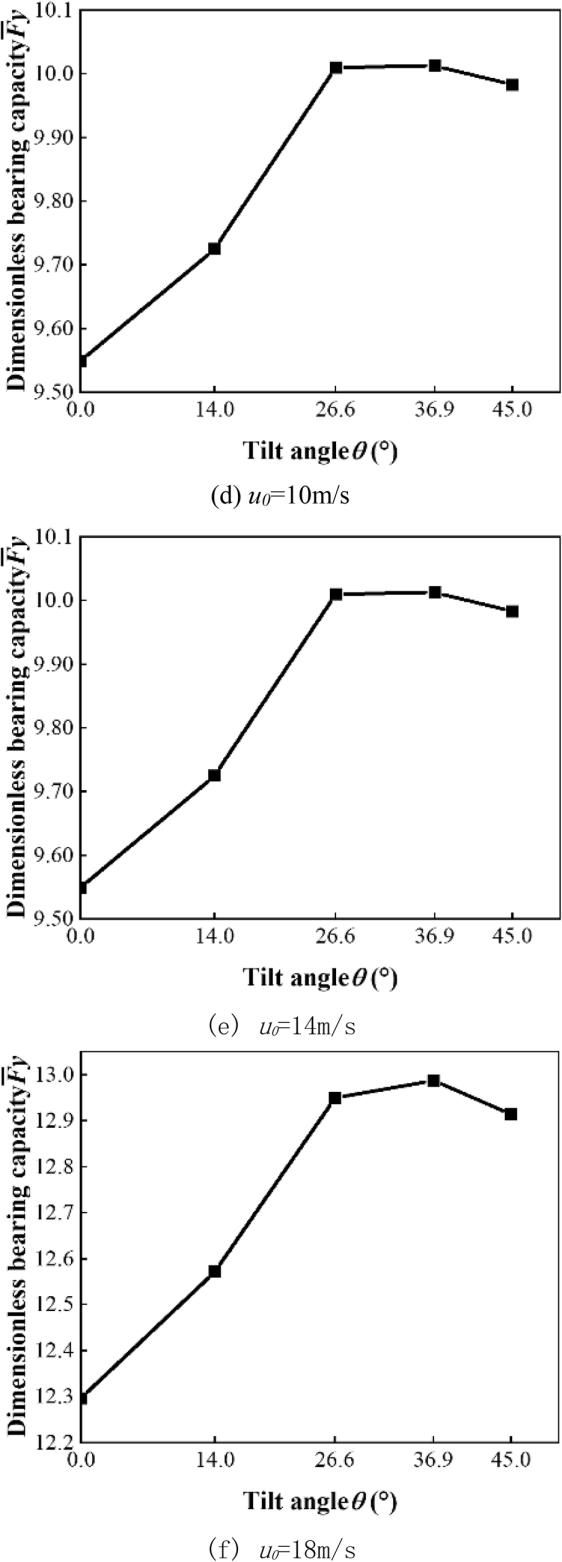
Bearing capacity.

Figure 6a shows the dimensionless bearing capacity of the square texture array and three parallelogram texture arrays with an inclination of 26.6°. With the increase in speed, the dimensionless bearing capacity of all pattern texture arrays gradually increases, and the difference in bearing performance between different pattern arrays is obvious. Within the speed range studied in this paper, the bearing capacity of the single-symmetric orientation texture array is always the best.

Figure 6b–f show the dimensionless bearing capacity of a asymmetrically oriented texture array with different tilt angles under different speed conditions.It can be seen from the figure that under various speed conditions, the asymmetric orientation array has an optimal tilt angle to make the dimensionless bearing capacity the highest. As the speed increases, the optimal tilt angle tends to increase. When the speed is 18 m/s, the optimal tilt angle of the asymmetrical array increases from 26.6° to 36.9°. This is because the increase in speed will increase the suppression of the dynamic pressure effect between the front and rear textures, and the greater the inclination angle of the parallelogram, the more conducive to staggering the low- pressure zone at the entrance of the texture and the high-pressure peak zone at the exit in the flow direction. The high-pressure zone of the texture outlet extends backward in the direction of flow, thereby increasing the range of the high-pressure zone.

### Analysis of experiments results

The interaction between textures can change the pressure distribution and flow field of the fluid domain to a greater extent, which is the main reason for the aggravation of cavitation. Different microtexture depth, arrangement, angle, shape and other factors will change its pressure distribution and flow field.

#### Formation principle of cavitation bubbles

**Figure 7:**
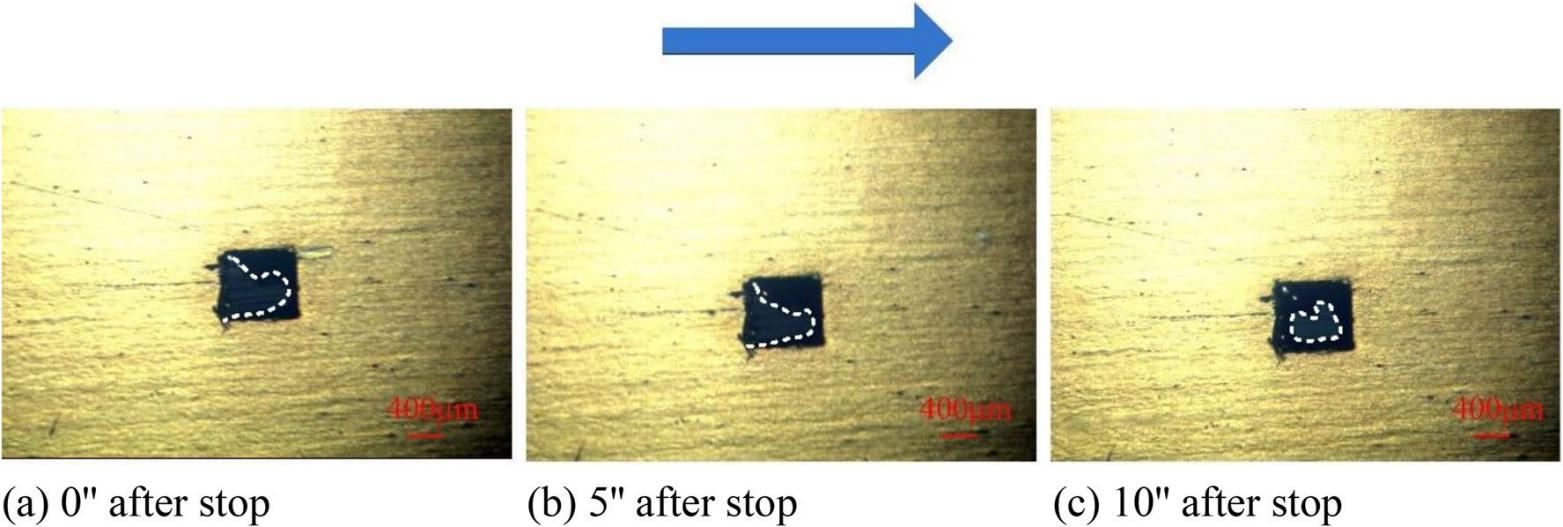
Cavitation bubble diagram after shutdown.

In^17^, the cavitation of lubricating oil is considered to be divided into gas cavitation and vapor cavitation. Typically, lubricating oils contain dissolved air with a saturation pressure at atmospheric levels. When the local liquid pressure is lower than the saturation pressure, the dissolved gas will escape from the solution, forming an air cavity. If the liquid pressure continues to decrease to the vapor pressure, the lubricating oil may boil at ambient temperature, forming vapor cavitation. Therefore, to further explore the cavitation type and bubble composition of texture-induced cavitation, the state of cavitation bubbles was continued to be observed after the experiment is stopped, as shown in Fig. 7.

It can be seen from the figure that when the rotating table stops, the bubbles in the texture do not disappear instantly, but gradually separate from the texture entrance, and converge into a whole bubble suspended inside the texture. This shows that under the conditions of this experiment, the main reason for the generation of cavitation bubbles is gas cavitation because the oil vapor generated by steam cavitation can be absorbed instantaneously, while the gas separated from the lubricating oil takes a long time to dissolve.

The behavior of texture-induced cavitation bubbles is a dynamic equilibrium process. First, the rotation of the lower sample makes a local pressure drop zone formed at the entrance of the texture, and the air precipitated in the lubricating oil is continuously accumulated after the formation of cavitation gas nuclei, and the air bubbles visible to the naked eye are gradually formed. Then, due to the shearing and dragging effect of the rotation of the lower sample on the lubricating oil and the air bubbles, the cavitation bubbles gradually extend downstream, and the flow of the lubricating oil will bring more dissolved air, and the continuously precipitated gas makes the air bubbles big. Finally, when the precipitation of lubricating oil gas and the dissolution of gas reach equilibrium, stable cavitation bubbles are formed.

#### Experiments on micro-textured arrays of different shapes

The fixed load is 10 N, and the experiment is carried out under different speed conditions to compare the friction coefficient of the non-textured sample and five kinds of texture samples of different shapes. The five texture arrays of different shapes can improve the lubricating performance of the friction pair compared with the non-textured samples, and their friction coefficients are all reduced.

When the speed is 6 r/min (0.019 m/s), the friction coefficient of samples are not much different. When the speed is low, the oil film of the friction pair has not been fully formed, and the texture has little effect on the improvement of the oil film bearing capacity. When the speed is greater than 18 r/min (0.057 m/s), the lubricating performance of the sample with the convergent trapezoidal texture is always the smallest. When the speed is 24 r/min(0.076 m/s), the cavitation pictures of texture arrays with different shapes are shown in Fig. 7. It can be inferred from the Figure that cavitation has occurred on the surface of the textured samples of various shapes. However, the different texture distribution positions will cause the pressure distribution around the texture to be different, and the cavitation bubbles caused by the texture will also be significantly different. The textures located downstream of the texture array are more prone to drag out cavitation bubbles. The reason is that these textures are closer to the pressure outlet and the surrounding oil film pressure is relatively low. In addition, comparing the cavitation bubbles induced by texture arrays of different shapes, it can be seen that the convergent trapezoidal texture array produces the least bubbles, while the divergent trapezoid texture induces the most cavitation bubbles.

#### Experiments on micro-textured arrays with different distribution patterns

**Figure 8:**
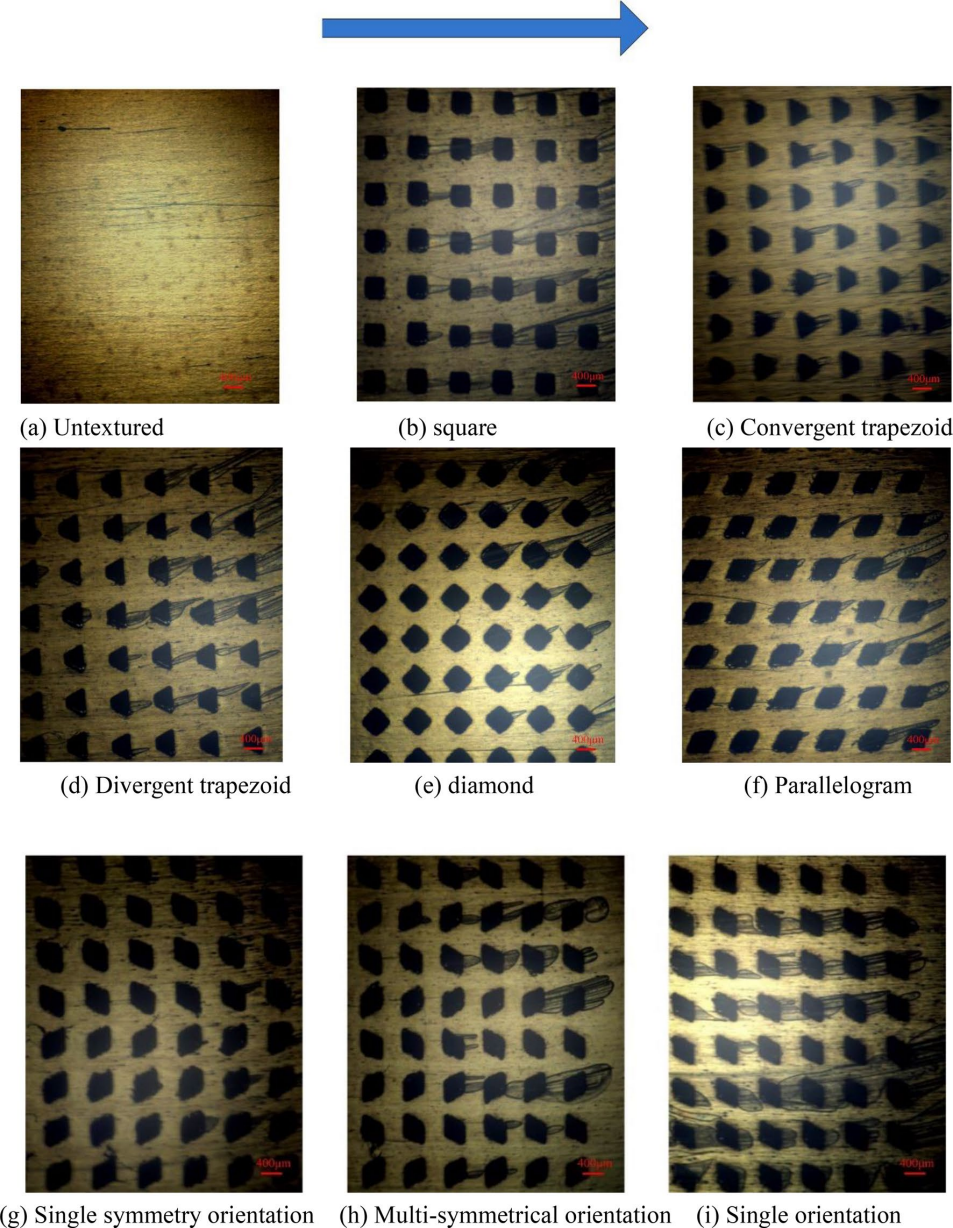
Cavitation images.

Figure 8 shows the cavitation state of three different parallelogram texture array samples at speed of 24 r/min. It can be seen from the figure that only a small part of the textures in asymmetric orientation texture array hascavitation bubbles overflowing. In a texture array with multiple symmetrical orientations, there are certain cavitation bubbles at the rear end of the outlet. The cavitation effect induced by the single orientation texture array is the most severe, which is due to the convergence of the cavitation bubbles of the upstream and downstream textures, forming a larger cavitation area. Comparing the pressure distribution of the texture array with different modes in the previous simulation, it can be seen that the area where the oil film pressure is higher is less likely to form cavitation bubbles.

#### Experiment on the oil film thickness of different pattern micro-textured arrays

**Figure 9:**
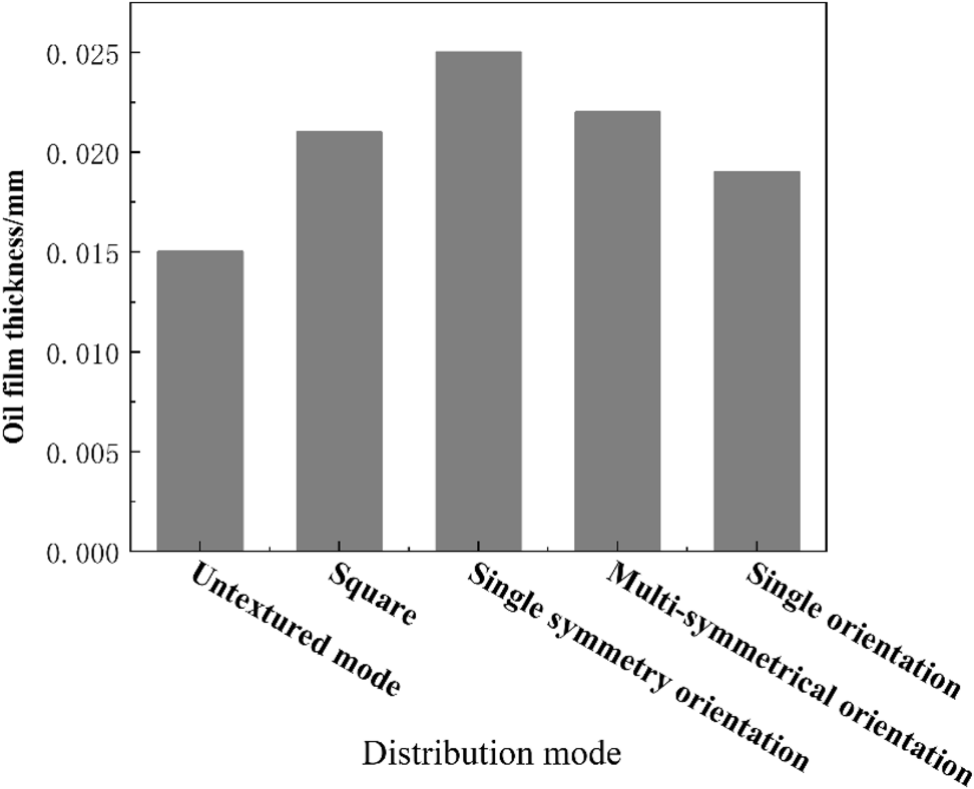
Oil film thickness of texture array with different patterns.

In order to further analyze the mechanism of the improvement of tribological performance by texture arrays of different patterns, the laser displacement sensor is used to estimate the oil film thickness. It can be seen from Fig. 9 that compared with the non-textured sample, the oil film thickness of other texture modes all have increased. Combining the previous simulation analysis, combined with the previous simulation analysis, it is found that the oil film bearing capacity of the textured array is directly proportional to the oil film thickness. This conclusion is consistent with the literature. The increase of the oil film thickness will reduce the friction coefficient of the sample and enhance the lubricating performance. Thus, the increase in the thickness of the oil film means that the direct contact area of the upper and lower sample is reduced, which makes the lubrication more sufficient.

## Conclusions

Based on the calculations and experiments, conclusions are drawn as follows:

1. Cavitation is one of the main reasons for microstructure to improve the bearing capacity of oil film.
2. The micro-textured array simulation results show that the single-symmetric orientation texture array is most conducive to the improvement of oil film bearing capacity, and the bearing effect is the best when the texture inclination is 26.6°. The interaction between the textures can significantly change the pressure distribution and flow field of the fluid distribution domain.
3. Experimental results show that surface textures can improve the lubrication effect of the friction pair, mainly because the micro-textured array improves the bearing capacity of the oil film and increases the thickness of the oil film.
4. The friction coefficient of the asymmetrically oriented textured sample is 29.4% lower than that of the untextured sample, and the lubrication performance is the best.

## References

[1] Yu H, Wang X, Zhou F (2010). Geometric shape effects of surface texture on the generation of hydrodynamic pressure between conformal contacting surfaces. Tribol. Lett..

[2] Woloszynski T, Podsiadlo P, Stachowiak GW (2015). Efficient solution to the cavitation problem in hydrodynamic lubrication. Tribol. Lett..

[3] Yang M, Liangcai Z (2016). Modeling and optimization of cavitation on a textured cylinder surface coupled with the wedge effect. Tribol. Int..

[4] Shi X, Ni T (2011). Effects of groove textures on fully lubricated sliding with cavitation. Tribol. Int..

[5] Etsion I (2004). Improving tribological performance of mechanical components by laser surface texturing. Tribol. Lett..

[6] Gropper D, Wang L, Harvey TJ (2016). Hydrodynamic lubrication of textured surfaces: A review of modeling techniques and key findings. Tribol. Int..

[7] Hamilton DB, Walowit JA, Allen CM (1966). A theory of lubri-cation by microirregularities. J. Basic Eng..

[8] Brizmer V, Kligerman Y, Etsion I (2003). A laser surface textured parallel thrust bearing. Tribol. Trans..

[9] Wang XL, Kato K, Adachi K, Aizawa K (2003). Loads carrying capacity map for the surface texture design of SiC thrust bearing sliding in water. Tribol. Int..

[10] Putignano C, Scarati D, Gaudiuso C (2019). Soft matter laser micro-texturing for friction reduction: An experimental investigation. Tribol. Int..

[11] Toshikazu N (2008). Micro-textures in concentrated conformal-contact lubrication: Effects of texture bottom shape and surface relative motion. Tribol. Lett..

[12] Hongyan R, Jianjunl L, Huil S, Xiaol W, Jianningl'Z D (2009). Research on hydrodynamic lubrication of different surface texture. China Mech. Eng..

[13] Wang X, Kato K, Adachi K (2003). Loads carrying capacity map for the surface texture design of SiC thrust bearing sliding in water. Tribol. Int..

[14] Yu H, Deng H, Huang W (2011). The effect of dimple shapes on friction of parallel surfaces. ARCHIVE Proc. Inst. Mech. Eng. Part J J. Eng. Tribol..

[15] Nanbu T, Ren N, Yasuda Y (2008). Micro-textures in concentrated conformal-contact lubrication: Effects of texture bottom shape and surface relative motion. Tribol. Lett..

[16] Wang X, Adachi K, Otsuka K (2006). Optimization of the surface texture for silicon carbide sliding in water. Appl. Surf. Sci..

[17] Shen C, Khonsari MM (2015). Numerical optimization of texture shape for parallel surfaces under unidirectional and bidirectional sliding. Tribol. Int..

[18] Siripuram R, Stephens LS (2004). Effect of deterministic asperity geometry on hydrodynamic lubrication. J. Tribol..

[19] Caramia G, Carbone G, De Palma P (2015). Hydrodynamic lubrication of micro-textured surfaces: Two dimensional CFD-analysis. Tribol. Int..

[20] Liu D, Yan Z, Yan X (2019). The simulation of asymmetric micro-textures on the lubrication characteristics of sliding friction pairs. IOP Conf. Ser. Earth Environ. Sci..

[21] Jiang S, Ji H, Wang T (2020). Enhanced understanding of leakage in mechanical seals with elliptical dimples based on CFD simulation. Ind. Lubr. Tribol..

[22] Brajdicmitidieri P, Gosman AD, Ioannides E (2005). CFD analysis of a low friction pocketed pad bearing. J. Tribol. Trans. Asme.

[23] Profito FJ, Vlădescu S, Reddyhoff T (2017). Transient experimental and modelling studies of laser-textured micro-grooved surfaces with a focus on piston-ring cylinder liner contacts. Tribol. Int..

[24] Wang T, Huang W, Liu X (2014). Experimental study of two-phase mechanical face seals with laser surface texturing. Tribol. Int..

[25] Qiu Y, Khonsari MM (2011). Experimental investigation of tribological performance of laser textured stainless steel rings. Tribol. Int..

[26] Wahl R, Schneider J, Gumbsch P (2012). In situ observation of cavitation in crossed microchannels. Tribol. Int..

[27] Bai L, Meng Y, Zhang V (2016). Experimental Study on Transient Behavior of Cavitation Phenomenon in Textured Thrust Bearings[J]. Tribol. Lett..

[28] Wang W, He Y, Li Y (2018). Investigation on inner flow field characteristics of groove textures in fully lubricated thrust bearings. Ind. Lubr. Tribol..

